# The Japanese Encephalitis Antigenic Complex Viruses: From Structure to Immunity

**DOI:** 10.3390/v14102213

**Published:** 2022-10-08

**Authors:** Baldeep Khare, Richard J. Kuhn

**Affiliations:** 1 Department of Biological Sciences, Purdue University, West Lafayette, IN 47907, USA; 2 Purdue Institute of Inflammation, Immunology and Infectious Disease, Purdue University, West Lafayette, IN 47907, USA

**Keywords:** Japanese encephalitis serogroup, Japanese encephalitis virus, Murray valley encephalitis virus, West Nile virus, Usutu virus, envelope protein, cross-reactivity

## Abstract

In the last three decades, several flaviviruses of concern that belong to different antigenic groups have expanded geographically. This has resulted in the presence of often more than one virus from a single antigenic group in some areas, while in Europe, Africa and Australia, additionally, multiple viruses belonging to the Japanese encephalitis (JE) serogroup co-circulate. Morphological heterogeneity of flaviviruses dictates antibody recognition and affects virus neutralization, which influences infection control. The latter is further impacted by sequential infections involving diverse flaviviruses co-circulating within a region and their cross-reactivity. The ensuing complex molecular virus–host interplay leads to either cross-protection or disease enhancement; however, the molecular determinants and mechanisms driving these outcomes are unclear. In this review, we provide an overview of the epidemiology of four JE serocomplex viruses, parameters affecting flaviviral heterogeneity and antibody recognition, host immune responses and the current knowledge of the cross-reactivity involving JE serocomplex flaviviruses that leads to differential clinical outcomes, which may inform future preventative and therapeutic interventions.

## 1. Introduction

Members of the genus flavivirus within the family *Flaviviridae* cause a substantial global burden of disease and mortality each year and pose a constant threat for future outbreaks. Yet, treatment for human flavivirus infections is lacking [1]. Depending on the type of vector involved in virus transmission, these arthropod-borne animal viruses, or arboviruses, are broadly divided into three groups: the mosquito-borne flaviviruses, the tick-borne flaviviruses and a third group with yet unidentified vectors. Historically, members of the family *Flaviviridae* were classified based on serological assays such as neutralization tests, hemagglutination inhibition assays, complement fixation and immunodiffusion [2,3]. Eight antigenic groups or serocomplexes in the genus flavivirus within the family *Flaviviridae*, classified on the basis of serological assays, have been described [4]. The tick-borne encephalitis antigenic group comprises members such as Omsk haemorrhagic fever virus, Russian spring–summer encephalitis virus (RSSE), Louping Ill virus, Kyasanur forest disease virus, Langat virus and Powassan virus. The Japanese encephalitis (JE) serocomplex includes Murray valley encephalitis virus (MVEV), Japanese encephalitis virus (JEV), West Nile virus (WNV), Kunjin virus (KUNV), Usutu virus (USUV), Kokobera virus, Alfuy virus and St. Louis encephalitis virus (SLEV). The four dengue serotypes form the Dengue serocomplex and the Spondwenii serocomplex includes Zika virus (ZIKV) and Spondwenii virus (SPOV). Yellow fever virus (YFV) forms a distinct serogroup. Genome sequencing and subsequent genomic and phylogenetic quantitative and bootstrapping analyses using pair-wise nucleotide sequence identity and clustering reveal the genetic relatedness of newly identified member strains and inform parameters influencing virus evolution, transmission and discovery [5,6]. A second classification system developed by Kuno et al. [5], defining fourteen clades (I–XIV), relies on the nucleotide and amino acid sequence identity of certain genes in the viruses. Similar to the serologic classification, the mosquito-borne viruses form a distinct cluster. Of these, the members of the Japanese encephalitis serocomplex discussed in this review, MVEV, JEV, WNV, KUNV and USUV, fall under clade XIV; DENV serotypes form clade IX; ZIKV and SPOV belong to clade X; the Nataya serocomplex members Bagaza virus, Tembusu and Israel meningo-encephalitis virus are part of clade XI; and YFV (Asibi) is part of clade VII [5,7]. These serological and phylogenetic relationships among flaviviruses provide a framework for understanding the host-immune interplay of viruses within and between antigenic complexes and flavivirus biology. For instance, antigenic classification correlates with the vector species involved in transmission; for the JE serogroup, this is largely the *Culex* spp., although other vector species are known to transmit these viruses as discussed below (Table 1) [4].

Arboviruses are zoonotic agents that transmit disease from vertebrate hosts (wild animals) to human beings via arthropod vectors such as mosquitoes and are maintained in the environment in zoonotic transmission cycles [15,16]. In the enzootic or sylvatic cycle, the female mosquito vector feeds on the infected vertebrate host that acts as the reservoir; when the virus replicates and infects the mosquito, the latter transmits the amplified virus to the next vertebrate via its salivary glands during a subsequent blood meal. Vertebrate reservoirs are typically birds, small wild animals or non-human primates; these are the natural hosts of the virus, and frequently transmission does not result in disease. In the epizootic or rural cycle, domestic animals are infected by either a primary or accessory vector which leads to outbreaks in the animal population. When humans are in close proximity, the virus can get transmitted to humans via the vector as well (urban or epidemic cycle). In the case of sufficient viremia, there is enough virus amplified to infect a new mosquito vector; otherwise, humans are dead-end hosts and do not perpetuate the virus transmission in the population. Non-vector modes of flavivirus transmission are less common and these have been described for DENV, JEV, WNV and ZIKV in humans and animals. Examples include transmission via organ transplantation, blood transfusion, oro-nasal secretions and horizontal transmission, and an uncommon, atypical case of WNV transmission via lactation [17,18,19,20,21]. Isolation from non-mosquito vectors such as ticks with transmission has been reported for WNV [22], as well as viral transmission in the absence of mosquito vectors in farmed crocodiles [23]. Sexual transmission of most flaviviruses in humans is uncommon. However, sexual and vertical transmission are established routes of ZIKV infection in humans [24]. Due to the overlapping ecological and geographical distribution of vectors and amplifying hosts, transmission of more than one virus by a single vector, simultaneously or consecutively, is feasible. Therefore, virus particles can adapt and survive the temperature and cellular milieu of multiple host and vector species and thrive in a broad diversity of micro-environments and tissues. Furthermore, multiple sequential infections with the same JE serocomplex virus or with a second flavivirus of the same or different serogroup can influence the host immune response and disease outcomes.

## 2. Distribution and Disease

**Figure 1:**
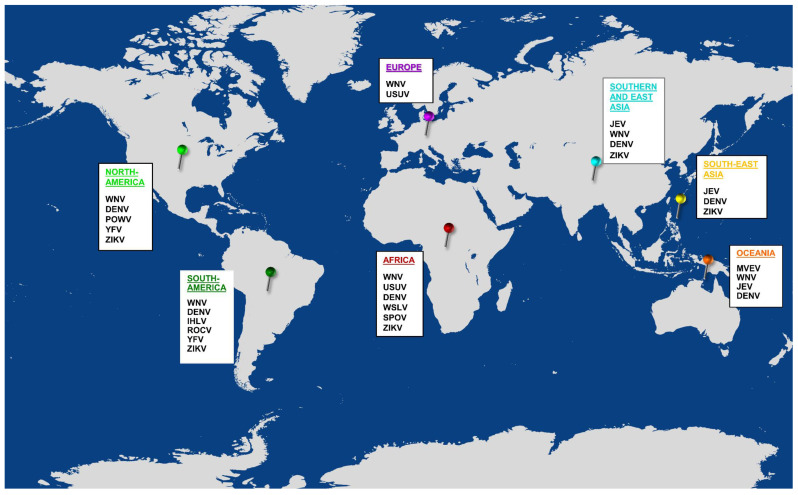
Global distribution of flaviviruses. Multiple flaviviruses co-circulate in most continents; of these, at least one virus belongs to the JE serocomplex. Co-circulation of more than one JE serocomplex virus occurs in Africa, Europe, Asia and Australia. The figure was generated using an online tool, URL: https://mapchart.net. (DENV: Dengue virus; IHLV: Ilheus virus; JEV: Japanese encephalitis virus; MVEV: Murray valley encephalitis virus; POWV: Powassan virus; ROCV: Rocio virus; SPOV: Spondwenii virus; USUV: Usutu virus; WSLV: Wesselsbron virus; WNV: West nile virus; YFV: Yellow fever virus; ZIKV: Zika virus).

JE serogroup viruses circulate in both temperate and tropical zones, with an expanse corresponding to a population of over three billion people (Figure 1). This wide distribution can be attributed to evolution, high genetic diversity, emergence and re-emergence of strains and natural spread of the virus due to vector proliferation and avian migration [25,26,27]. Distribution of specific strains is directly linked with climate change [25,28]. Based on the geographical distribution and phylogenetic analyses of whole genome, the NS5 or the envelope (E) gene, genotypes 1–4 (G1–4), genotypes I–V (GI–V) and eight lineages each have been described for MVEV, JEV, WNV and USUV, respectively (Table 2). 

The geographic distribution of JEV includes countries in Asia and Southeast Asia. JEV causes an estimated annual burden of 68,000 cases every year; the global disease burden is unknown but is estimated to be approximately 20,000 fatalities. JEV encephalitis has a mortality rate of up to 30% and survivors can have permanent neurological or psychological sequelae. GIII used to be the dominant genotype in temperate zones, and it was associated with human outbreaks in the past. GI represents the dominant genotype of JEV in the world [25]. MVEV is spread across the Northern Territory, Western Australia and the south-eastern region in Australia. It infects humans and animals from time to time, causes meningitis and encephalitis in rare cases and varying degrees of brain dysfunction. GI is the dominant strain across all areas of distribution, whereas some others like GIB (a sub-strain of GI) and GII are more restricted [31]. WNV is one of the most widespread viruses in this antigenic complex and has caused outbreaks in many countries. By 2002, WNV lineage I strain, NY99, had spread across the USA with the emergence of new, more virulent strains [35]. WNV lineage II, with a dissemination in Europe around 2008–2009, is currently the dominant strain in the region and responsible for multiple epidemics in humans and animals. Both WNV lineages cause a neuro-invasive disease in 1% of the infected human population [35]. USUV is currently restricted to Europe and is mostly asymptomatic in humans [46]. Two cases of human illness have been reported from Africa, whereas European strains are the cause of severe illness in humans only in rare cases. 

The WNV and JEV distribution overlaps with that of other heterotypic flaviviruses such as ZIKV and DENV in parts of Asia and South America, whereas WNV and JEV overlap in Asia. In Europe, WNV and USUV share a large geographic distribution, as well as many vectors and amplifying hosts. The overlap of geographic distribution has implications for disease diagnosis and control and host immune response (discussed below). A multitude of factors add to the ecological interplay, such as the genetic diversity of the virus, co-circulation of multiple strains and viruses and distinct pathology displayed by different lineages. 

## 3. Virus Morphology and Host Interplay

Flavivirus pathogenicity, extensively studied using molecular and structural biology together with animal models in ZIKV as well as in the hemorrhagic DENV, is intricately linked with virus assembly, maturation, host cell interactions, the immune response and membrane fusion [47,48,49,50,51]. Virus morphology affects disposition of structural proteins and surface residues and hence impacts interactions with host receptors and immune cells [52,53]. Previous reports are recommended for an in-depth review of the current understanding of the flavivirus life cycle, domain organization and the structural biology of the envelope protein (E), the premembrane protein (prM), the capsid protein (C), and structures of complexes of these proteins with receptors and antibodies [52,54,55]. Fundamental structural understanding stemming from investigations in DENV, WNV, ZIKV, TBEV and JEV structures suggests that many molecular interactions and mechanisms may share some similarity within flaviviruses [56].

### 3.1. Distinct Structures Correlate with Specific Stages of the Viral Life Cycle

**Figure 2:**
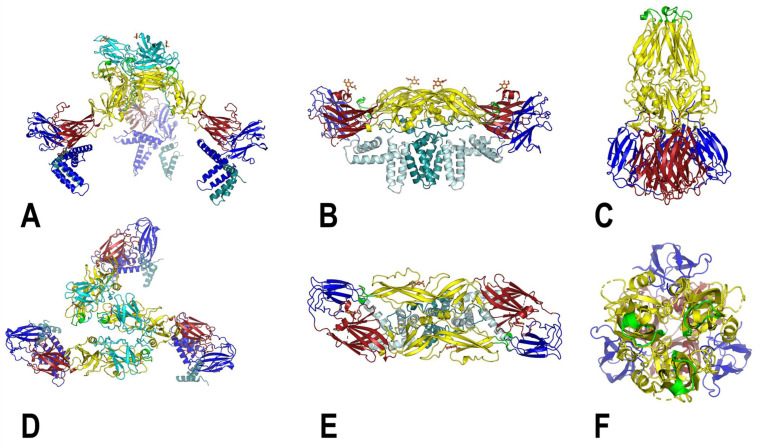
Models of the flavivirus structural proteins corresponding to various stages in the virus life cycle. (**A**–**C**): Immature DENV1 trimer, mature USUV dimer and the fusogenic DENV hairpin conformation, respectively. Corresponding top views are depicted in (**D**–**F**), respectively. For the immature E trimer and the E dimer, the E-stem region is also shown. The models are color coded as follows: DI (red), DII (yellow), DIII (blue), fusion loop (green), glycosylation sites (orange) and prM (cyan); for (**B**,**E**), the E-stem is in light blue and M protein is in teal.

Within infected cells, flavivirus interaction with host proteins in the endoplasmic reticulum (ER) alters its morphology at specific sites where replication of the RNA genome and assembly of new virions takes place [57,58,59]. Immature flavivirus particles are roughly 60 nm in diameter, assemble at neutral pH and bud into the ER lumen [60]. An immature flavivirus is composed of the structural proteins, E, prM and C, and the viral RNA genome; the transmembrane regions of E and prM are embedded in a lipid envelope that surrounds the nucleocapsid [60,61]. Sixty heterotrimers of E and prM form ‘spikes’ that project out from the virus surface; the trimer is positioned at the quasi-threefold of the asymmetric subunit, and the fusion loop (FL) at the distal end of the E:prM trimer is protected by prM (Figure 2) [60,61,62,63,64]. However, FL in the immature virion can be targeted by antibodies and must be accessible [65,66]. Immature virus is non-infectious by virtue of the presence of prM, which shields the fusion loop (FL) of E and prevents membrane fusion [67,68,69]. Low resolution, asymmetric cryo-EM reconstruction suggests that immature virions bud with an eccentrically positioned nucleocapsid core relative to the outer icosahedral glycoprotein shell [70]. The electron density present at the base of the spike formed by transmembrane domains of three E/prM monomers and positioned between the inner leaflet and the nucleocapsid core was observed in immature ZIKV structures; this corresponds to a single capsid protein which interacts with the glycoprotein transmembrane domain [71,72,73].

As the immature virus traverses the acidic compartments of the trans-Golgi network, conformational changes in E and concomitant processing of prM facilitate flavivirus maturation. Acid-induced conformational rearrangement leads to the formation of an icosahedral shell of 90 E:prM antiparallel heterodimers that lie ‘flat’ on the virus surface, resulting in a smooth outer appearance; prM is subsequently cleaved into ‘pr’ and ‘M’ proteins by the host furin enzyme and ‘pr’ is released when exposed to the neutral pH of the extracellular milieu [62,68,74,75,76]. The mature flavivirus is 50 nm in diameter and displays the ‘herringbone’ array of E rafts, a hallmark of the mature virion (Figure 2) [77]. Mature flavivirus is infectious, multiple molecular determinants within E confer specific tropism and subsequent virus entry in the host cells is mediated via attachment factors [78,79] which represent a primary target for host antibodies. Multiple high-resolution structures showed the absence of capsid density near the E/M transmembrane domain and between the lipid layer and nucleocapsid core, which suggests a rearrangement of C in the mature virus [62,71,74,75]. More significant structural changes occur in the M-TMD than in the E-TMD between the immature and mature forms of flavivirus [80].

Infectious, assembled flavivirus particles are an assortment of mature and mosaic particles that interact with multiple, often redundant, host cell surface receptors/attachment factors to gain entry into a cell [81]. However, purified JE serocomplex viruses expressed in mammalian cells are relatively less heterogeneous than those observed for DENV, which may possibly be a type-specific difference. Known flavivirus receptors/attachment factors, including those for JE serocomplex viruses, fall under the broad classification into families of proteins such as C-type lectins or CLRs (e.g., dendritic cell-specific ICAM-3 grabbing non-integrin or DC-SIGN, a homologue of DC-SIGN named DC-SIGNR, C-type lectin domain family 5 member A or CLEC5A), integrins (e.g., αVβ3, αVβ5, αVβ1), phosphatidyl serine receptors of the TIM/TAM families (T cell immunoglobulin mucin domain; Tyro3, Axl and Mer), heat-shock proteins (e.g., heat shock proteins HSP70 and HSP90, heat shock cognate HSC70) as well as tight junction proteins (Claudin-1), heparin sulfate proteoglycans (HSPGs) and glycosaminoglycans (GAGs), laminin receptors and natural-killer-cell-activating receptor NKp44 [82,83,84,85,86,87]. Interactions with receptors/attachment factors affect cellular and tissue tropism and hence disease manifestation. However, successful cellular infection is a multiple-step process (attachment, recognition, binding, viral entry and virus internalization) that requires both receptors/attachment factors and additional host factors that facilitate viral replication in a permissive cell [87]. For the JE serocomplex, some receptors/attachment factors have been identified using in vitro experiments, but their role in viral entry and the molecular mechanisms of highly specific receptor binding are unclear [50,82]. For instance, JEV is neuroinvasive and known to breach the BBB; it infects pericytes, glial cells and developing neuronal cells, and utilizes CLRs, TIMs, HSPG and GAG, integrins and heat-shock proteins as receptors/attachment factors, yet the JEV receptor responsible for viral entry in the central nervous system remains elusive [50,82,88]. Virus–receptor interactions greatly depend on particle morphology and can trigger cellular changes that facilitate virus internalization and clathrin-mediated endocytosis. 

Extensive reviews have described flavivirus characteristics and structural heterogeneity that pertain to viral entry (discussed in Section 3.2) [50,81,87]. Molecular determinants on E confer binding to various receptors/attachment factors, but these are not always well defined. Specific amino acid residues in JEV, MVEV and WNV can drive viral entry and these reside on different domains of the E protein [89]. The surface properties of the E protein, in particular the presence of Lys-rich residues in the DI or DIII domains, confer positive charge, which drives the interaction with the negatively charged GAGs in JEV, WNV and MVEV [90,91], and similarly localized Lys residues in some USUV strains may confer preferential GAG binding [75,92]. In the mature, infectious form, these receptor-binding determinants would need to be exposed for interactions, as is indeed the case for some interactions, while, conceivably, some E elements that participate in interactions may become accessible only during intermediate stages, possibly post-attachment. Known findings on CLR and integrin interactions attest to these differences, for instance, glycosylation at the Asn-154 site on the glycan loop is a marker of virulence in JEV, WNV and MVEV and mediates differential binding to CLRs [93]. On the other hand, while integrins have been implicated in WNV and JEV entry, the role of the RGD motif in mediating WNV entry has been inconclusive from in vitro studies and high-resolution details showed the RGD motif in USUV to be unavailable for interactions [74,75,94,95]. DENV and USUV are the only two flaviviruses that contain a second glycosylation site, at Asn-67 and Asn-118, respectively. All serotypes and strains within the two respective antigenic complexes are known to possess the second glycosylation site; however, other JE serocomplex members including WNV, JEV and MVEV lack this site, which prompts a question of evolutionary conservation and suggests some entry/fitness advantage of the DII glycan site for DENV and USUV. Structural knowledge of E protein with receptors/attachment factors revealing atomic-resolution details of the virus–host interface in complexes is lacking [96]. An integrated structural approach involving X-ray crystallography and cryo-electron microscopy (cryoEM) may be essential to fully decode the structural basis of flavivirus recognition and binding of cognate attachment factors or receptors as well as define the molecular determinants and key interactions involved in viral entry. 

Once endocytosed in a newly infected cell, an irreversible, acid-induced conformational change occurs in the mature virus that leads to the realignment of inter-domain interactions and dissociation of the E:M heterodimers; the repositioning of DII, DIII and the E-stem; and subsequently the formation of sixty E:E:E homotrimers [64,97]. Formation of the acid-induced trimeric fusion-competent form is essential for fusion and a requisite prelude to the release of viral RNA into host cytoplasm [98]. E homotrimers expose three FLs at the distal end for insertion into the endosomal membrane (Figure 2) [99]. However, since the process is dynamic and transient, the intermediate states are often captured by complexing with antibody fragments. Complex formation of WNV with monoclonal antibody (mAb) E16, which blocks WNV membrane fusion by inhibiting E-trimer formation, followed by a drop in pH, arrests the virus in a pre-fusion state [100]. The WNV pre-fusion state reveals an expansion of the E shell, with a gap of 60 Å between the E and lipid layers. DENV post-fusion intermediates further detail the “open” and “closed” states that are on the continuum leading to fusion: in the “open state”, the FLs are inserted into the endosomal membrane but the DI-DII hinge angle is the same as that in the E:M heterodimer of mature virus, whereas in the “closed” state, additionally, E-stem helices and DIII are repositioned to facilitate E-stem “zippering” [99,101,102]. 

### 3.2. The Sources of Heterogeneity Are Multifactorial

Flavivirus particles produced in an infected cell are a heterogeneous mixture of mature, partially mature and immature virus particles [103,104]. This primarily results from inefficient furin processing of the prM, which has been observed primarily in DENV and JEV [105]. The mature and immature particles contain M and prM, respectively, whereas, in partially immature particles, both M and prM are present in the same particle and prM and E associate as a heterodimer that lies close to the viral surface [106]. The latter may represent an intermediate along the continuum of prM processing; indeed, structural studies confirmed that these particles contain features characteristic of both the mature and immature virions [106,107]. Mutational analysis of DENV prM residues at and around the furin cleavage site and chimeras containing prM segments from JEV, YFV and TBEV showed enhanced cleavage for the JEV chimera and delayed egress of the virions but no change in infectivity [108]. Cleavage efficiency and hence the degree of heterogeneity varies among flaviviruses and may also vary between viruses/strains within the JEV serogroup; for instance, purified SAAR-1776 USUV displayed mostly mature particles with a low number of immature particles and fewer partially immature particles. However, the visual appearance of particles, without further analysis with virometry or mass spectrometry, is insufficient to conclude the nature of heterogeneity [75,105,108]. The cell type used for virus production is important as mammalian cells result in less heterogeneity; however, it remains to be established whether the range of variation in heterogeneity might correlate with the pathogenicity of strains for specific hosts, within and between serogroups [103,109]. 

Fundamental knowledge of the structural biology of flaviviruses comes from investigations of DENV serotypes and their interactions with host components. However, in the broader context of the structural architecture of flaviviruses that is pertinent for pathogenesis, common features are shared while finer details may differ between type-specific viruses or strains. The morphology of the mature DENV virions spans a broad range: the particles include those with a ‘smooth’ (diameter of ~500 Å) appearance or a rough, ‘bumpy’ appearance (somewhat variable sizes with reported diameters between 360–550 Å), depending on the host infected or types of cells used for virus production, and a non-spherical structure referred to as the club-shaped particles [110,111,112]. Dengue virions change to the ‘bumpy’ morphology above 33 °C, and, therefore, at the physiological body temperature of the human host (37 °C), this form is expected to dominate, whereas in the mosquito vector (28 °C), *Aedes* spp. mosquitoes for DENV, the smooth form would predominate. This phenomenon of temperature-dependent particle expansion is referred to as viral ‘breathing’. Structurally, the ‘bumpy’ particle shows expansion of the protein shell and some structural rearrangements of the E protein domains compared to the smooth morphology. The protein shell containing E lies at a greater radius from the center of the particle whereas the radial distance of the lipid layer remains unchanged, protrusions of E domains I and III (DI and DIII) are observed between the five- and three-fold icosahedral axes, weakening of E dimer interactions at the icosahedral two-fold shifts the raft arrangement, and a hole is present at the icosahedral three-fold vertices that is surrounded by the DI and DIII domains [111]. Structural studies reveal that the temperature-dependent expansion observed in DENV is serotype-specific and is shown only by DENV2. 

Evidence for viral breathing in JE serocomplex viruses comes from functional studies on WNV (described below) using antibodies E60, E16 and E53, and Thr198 of WNV E (and Phe193 in DENV1) [113,114]. In the JE serogroup, cryo-EM structures of mature viruses have been determined for USUV, WNV and JEV (resolutions of 2.4, 3.1 and 4.3 Å, respectively) and for chimeras of Binjari virus with WNV and with MVEV (resolution of 2.9 and 3.7 Å, respectively), where WNV or MVEV structural proteins, respectively, form the icosahedral glycoprotein shell [74,75,115]. USUV structures represent the highest-resolution maps of a mature flavivirus solved to date using cryo-EM, and these are also the highest-resolution structures from the JE serogroup [75]. The maps reveal densities for three lipid sites; none of the other recent structures of mature flaviviruses reveal the presence of all three sites and this could be due, in part, to the higher number of particles used for the reconstruction and the biological component pertaining to the disposition of M helices in the membrane, which is most similar for ZIKV and USUV [75,116]. One of the two sites near the E-stem (referred to as ‘S2’) is also present in the flavivirus cryo-EM structures of ZIKV, SPOV and the chimera of Binjari virus and DENV; therefore, the two sites may be functionally distinct and S2 may represent an essential lipid interaction site across flaviviruses [62,74,75,116,117]. 

A non-spherical morphology for DENV, called the club-shaped particle, was recently described [118]. Morphologies with a head and a tail (HAT particles), albeit a slack tail, similar to club-shaped particles have been observed in some purified flavivirus samples where the number of HAT particles increased with time and were observed with a concomitant decrease in the number of spherical virions. However, these changes are differentially observed for flaviviruses (unpublished data). A structurally distinguishable subpopulation of USUV was also recently described where the differences were restricted to side-chain conformations of residues and the presence of an FL disulfide bond in one of the three monomers of the asymmetric subunit; the simultaneous existence of virions displaying different conformations in a sample and elucidation of their cryo-EM structures has been reported for other viruses [75,119]. This subpopulation represented about 33% of the sample size in USUV, comparable to the class III particles of the ‘bumpy’ DENV2 extracted for cryo-EM reconstruction, emphasizing that the presence of flavivirus subpopulations within a sample may have functional implications for host interactions [75,111].

### 3.3. Particle Architecture Affects Host Interactions

**Figure 3:**
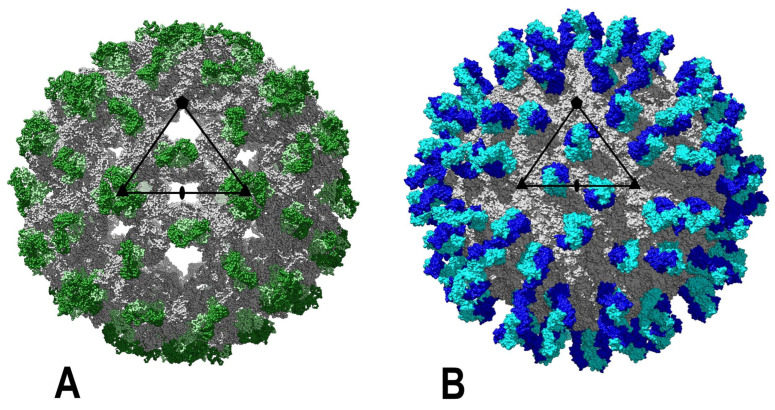
Models of some JE serocomplex flavivirus particles with bound Fabs. (**A**). Immature WNV particle with bound E53, which is cross-reactive and preferentially binds immature flavivirus particle but not the mature particles. The Fab chains are depicted in green and light green. (**B**). Mature WNV with bound CR4354, which binds two neighboring E molecules and neutralizes by blocking the conformational rearrangement essential for membrane fusion. Fab chains are shown in blue and light blue.

Mature and immature forms of flaviviruses, by virtue of the presence of prM, and the fusion-competent forms of the virus possess distinct particle architecture and hence display differences in the surface-exposed regions of the structural proteins; this has direct implications for antibody recognition and binding. Antibodies can target both structural forms of flaviviruses and antibodies that target all three domains as well as the DI–DII and DII–DIII hinge regions of E have been identified and structurally characterized for DENV [55]. The neutralization potency of the antibodies varies. Some antibodies target the same region in multiple flaviviruses, including DENV and ZIKV, such as the E dimer epitope (EDE)-recognizing monoclonal antibodies that crosslink the two E proteins in the homodimer [55,65]. EDE antibodies can be further classified into EDE-1 and EDE-2 antibodies, where only the latter require the Asn154 glycosylation on the adjacent E of the dimer for recognition. Furthermore, EDE antibodies can bind both mature and partially mature virion, exemplifying the interplay of antibodies and virion architecture. Binding of antibodies such as CR4354 and 14C10, specific for WNV and DENV1, respectively, results from recognition of quaternary epitopes on the E dimer of the mature virion; however, the occupancies of the antibodies on the flaviviruses vary, further specifying the residues recognized by different antibodies on mature virions (Figure 3) [55,120]. The maturation state, virion stability and “viral breathing” affect antibody recognition and hence virus neutralization in WNV and DENV [65]. For instance, DENV E-specific antibody 1A1D-2 binds to the mature virus after incubation at 37 °C as the epitope is not accessible at 4 °C [121].

Epitopes recognized by antibodies in the immature virus may become inaccessible when E monomers form dimers in the mature virus. A change in neutralization sensitivity and hence potency was observed in response to WNV maturation as a result of epitopes becoming less accessible in the mature virion [122,123,124]. Conversely, studies in MVEV revealed that prM in the immature particle conceals epitopes accessible in the mature particle and the prM-associated immature particles are more acid-resistant [125]. Another “cryptic” or inaccessible epitope on the immature virions is the fusion loop epitope (FLE) that is recognized by the WNV E53 antibody [126]. These antibodies bind immature virions, as well as the partially mature particles or particles undergoing viral “breathing”, all scenarios where FLE is exposed [65]. FLE–recognizing antibodies in DENV were shown to be strain- and DENV serotype-dependent, suggesting the need for evaluation of individual strains [127]. E53 preferentially recognizes partially mature WNV virions and fails to neutralize mature virus [126]. 

FL-recognizing antibodies tend to be weakly neutralizing and cross-reactive with other flaviviruses due to the strong conservation of the FL; however, Vogt et al. showed that weakly neutralizing WNV FL antibody E28 conferred protection in mice in vivo via effector activation and phagocytic activity even though E28 showed poor neutralization in vitro [128]. Vogt et al. further speculated that the protective effects of cross-reactive FL antibodies could be protective for secondary WNV infections in geographical areas where more than one flavivirus is circulating; this may be relevant for the observed protection recently reported for WNV lineage II in Europe in patients with pre-existing USUV immunity (discussed later). Immuno-dominance of FLE antibodies is also reported in other flaviviruses in studies using polyclonal sera from infected and vaccinated individuals [129]. While morphology affects infectivity and antigenicity [65,126], antibody binding can affect viral function. The flavivirus humoral response generates antibodies against E and prM; a peculiar feature of some prM antibodies is their ability to render immature particles infectious as these facilitate binding and internalization into cells containing Fcγ receptors [69,130].

## 4. JE Serocomplex Flavivirus Immune Response

### 4.1. Innate Immunity

Clinical manifestations of JE serocomplex virus infections in humans span a wide range, from asymptomatic prevalence or mild febrile illness to neuro-invasive disease and encephalitis. Severe neurological disease is more likely to afflict the elderly and immune-compromised individuals. Studies aimed at understanding the molecular basis of pathogenesis and the host immune response in these diverse scenarios, using in vitro and in vivo murine models such as mice with single or double knock-outs of effector molecules, their receptors or other components, helped identify cellular components and biomarkers critical for viral restriction and the spread of infection in peripheral tissues as well as identify factors that may contribute to neuro-invasive disease, immunopathology or exacerbation of outcomes toward severe disease [105,131,132]. In addition to the three structural proteins (E, C and prM), the flavivirus polyprotein encodes seven non-structural proteins (NS1, NS2A, NS2B, NS3, NS4A, NS4B and NS5) [53]. Protection emanating from the innate defense against these viral components generates a non-specific, broad-range response that is essential for viral restriction and clearance and limiting progression of the disease. Pattern recognition receptors (PRRs) on mammalian antigen-presenting cells, Langerhans, human primary keratinocytes and dermal dendritic cells in the skin detect pathogen-associated molecular patterns (PAMPs) such as double-strand viral RNA. Binding of viral components triggers a downstream signaling cascade, beginning with the activation of one or more of the three kinds of receptors: retinoic-acid inducible gene-I (RIG-I)-like receptors (RLRs); melanoma-differentiation-associated-gene 5 (MDA5) in the cytoplasm; and nucleotide oligomerization domain (Nod)-like receptors (NLRs) and Toll-like receptors (TLR; e.g., TLR3, TLR7 and TLR8) in endosomes [132,133,134,135]. Activation of downstream adapter molecules with kinase activity (e.g., NEMO, IKKα, IKKβ; TBK, IKKε) activates transcription factors (e.g., IRF3 and IRF7) and NF-κB in a cell-type-specific manner [132,133]. IRF1, IRF3, IRF5 and IRF7 all restrict WNV replication; however, IRF5 plays a non-redundant, immunomodulatory role in shaping the early immune response events via production of pro-inflammatory cytokines in the lymphoid tissues, but not type I interferons, and also affects the trafficking and activation of immune cells entering the draining lymph node [136]. IRF5 further adversely impacts early antibody response in mice. In vivo experiments revealed that IPS-1, a key adaptor in the RIG-I signaling pathway, and a transcription factor *Batf3* regulate inflammation via interactions with T cells [137,138]. Once translocated to the nucleus, each transcription factor induces the expression of specific genes, and, subsequently, the production of inflammatory cytokines (type I (IFN-α, IFN-β), type II (IFN-γ) and type-III (IFN-λ) interferons), and a multitude of interferon-stimulating genes (ISGs), which have antiviral effects as they restrict viral replication and dissemination in the host [105,133,139,140,141,142]. Eliciting production of interferons with a different virus was also shown to reduce WNV titers in vitro [143]. Secreted cytokines further interact with interferon receptors in virus-infected cells (autocrine or paracrine) and activate the Janus kinase/signal transducer and activator of transcription (JAK/STAT) pathway. The interactions of effectors of this pathway with interferon regulatory factors (IRFs) and binding to interferon-stimulated response elements triggers the expression of ISGs that eventually exert an antiviral state directly through effectors [144]. 

Known viral features that play a role in conferring neuro-invasiveness in WNV, as in many other flaviviruses, including those of JE serogroup, reside on the structural envelope protein, and N-linked glycosylation specifically is in the E protein [145]. In contrast, in USUV, N-linked glycosylation is present at Asn154 and Asn118 across strains, yet human USUV infections display a wide range of manifestation from asymptomatic presence with antibodies against the virus to meningo-encephalitis, suggesting that, in USUV, other features in the structural proteins or factors may facilitate neuroinvasion. The host milieu and components therein are also known to affect WNV neuro-invasiveness: the blood–brain barrier (BBB) is present at the blood-to-brain interface and is a physiologically and functionally distinct region made up of a vascular basal lamina; brain capillary endothelia cells (BCECs) characterized by the presence of tight junctions, adherens junctions and low vesicular traffic; and the neurovascular unit (NVU) consisting of pericytes, perivascular fibroblasts, glial cells and astrocytes [146]. Breaching the BBB may precede neuroinvasion and occurs via the compromised permeability of BCECs or the NVU, and in WNV infection, the underlying mechanisms of CNS entry and infection may involve cytokines like IL-6 and TNFα, semaphorin 7A, metalloproteinases, a ‘Trojan horse’ route utilizing infected immune cells, direct axonal retrograde transport, infection via the olfactory bulb and direct infection of cells of the NVU or neuron-to-neuron infection [134,147,148,149,150]. The neuroinvasion property differs across WNV strains and may involve variable mechanisms among WNV strains and between JE serocomplex viruses and is yet to be elucidated [151,152]. 

The innate immune response to WNV is essential for limiting viral dissemination to the CNS. In WNV-infected mice and patients with West Nile virus fever (WNF) and West Nile virus neuro-invasive disease (WNND), upregulation and elevated concentrations of a multitude of cytokines and chemokines were observed in sera and cerebrospinal fluid (CSF), with higher levels of pro-inflammatory cytokines in patients with WNND compared to those with WNF, and also during JEV infection [153,154,155]. These included markers of inflammation (such as IL1α, IL4, IFNα and TNFα), type 2 cytokines (such as IL4 and IL13) and IL10, which may be associated with the exacerbated immune response in patients with WNND. Neurological damage in WNND results from both neuro-inflammation and direct viral infection of the brain cells such as astrocytes, microglia and neurons. Studies in mouse models revealed an essential role for interferon γ (IFN-γ) in restricting viral replication, viral infection of peripheral tissues and the early onset of CNS infection. While the innate immune response is best described for WNV and investigated in JEV, these pathways are yet to be understood in-depth for USUV; however, animal models for in vivo and in vitro studies of USUV pathogenesis have been described [156]. Emerging research provides comparable insights into USUV and WNV pathogenesis and the immune response within a system: the use of distinct cellular tropism that involves specific receptors (langerin and DC-SIGN) and differential infection, replication and activation and the susceptibility of the innate immune response in different cell types such as Langerhans cells or human neural stem cells (hNSC) [157,158,159]. For instance, both WNV and USUV (strain Vienna_2001) induce a robust innate response in hNSC with high levels of type I and III IFNs and caspase-3, but WNV may be more efficient in evading the host immune response; the latter effect was also observed in dendritic cells [157,158]. JE serocomplex viruses, like other flaviviruses, have adapted multiple mechanisms to evade some innate immune response; therefore, these are not primary targets for therapeutic development [133,139].

### 4.2. Adaptive Immunity

The essential roles of CD4+ and CD8+ cells in modulation, control and protection in JE flavivirus infections have been described for JEV and WNV [105,142,160]. These involve viral clearance by CD4+ cells via its multitude effector functions including cytokine production, CD8+ response enhancement and maintenance of antibody response, and CD8+ cell-mediated lysis of infected cells by secretion of effectors such as Fas receptor, perforin or granzymes; however, the determinants of T cell immunity are better explored for WNV than for JEV or other JE serocomplex flaviviruses. For instance, in a mouse model of JEV encephalitis, the role of T helper cells was essential to maintain humoral immunity and counteract infection and lethality, and a robust CD8+ activation was marked by an increase in CD69 and CD25. However, the CD8+ response didn’t uniquely contribute to animal survival while the viral burden in the CNS of mice lacking this response was higher [161]. On the other hand, CD8+ is essential for clearance of WNV and in the CNS and recovery in mouse models [162], and variation in susceptibility was observed in mice deficient in Fas- or perforin-dependent cytolytic pathways between WNV lineage I and II, JEV and MVEV [161], which highlights the importance of elucidating a T cell response for specific flaviviruses of the same antigenic complex. Recent studies highlighted the important role of CD8+ cells in eliciting antibody-mediated protection in response to JEV vaccination in mice and in protecting JEV-infected mice via granule-mediated cytotoxic effects with a contribution of γ/δ TCR expressing T cells [163,164]. The T cell immune response in MVEV and USUV infections is less thoroughly defined. Some insights into the host T cell response to USUV infection comes from retrospective analyses of asymptomatic blood donors in WNV- or USUV-infected individuals, and a USUV-specific T cell response could be distinguished with high accuracy [165].

A significant protective defense is mounted by the host humoral immunity that generates neutralizing antibodies targeted against non-structural and structural proteins, including NS1, NS3, NS5, prM and the structural protein E on the virus surface. A number of epitopes of flavivirus E proteins have been mapped and some of the most potent neutralizing antibodies in flaviviruses like DENV and ZIKV are known to be directed against DIII and its lateral ridge comprising strand A, BC-, DE- and FG-loops [65]. Both B and T cell epitopes have been determined for JE viruses but fewer have been for JEV and MVEV than WNV, and a much lower number of epitopes has been identified for JE serocomplex viruses compared to DENV2 [166,167]. On the E protein, B cell epitopes in WNV map to the DIII lateral ridge, DI lateral ridge, DI linker region, DII hinge interface, DII dimer interface, DII central interface, DII lateral ridge and DII [113,168,169]. Unlike DENV, WNV neutralization is not dominated by DIII-lateral-ridge-directed antibodies, as studied in horses and humans and for the WNV strains associated with recent outbreaks [168,170,171], and includes a large repertoire directed at the fusion loop (discussed below). While epitopes are identified using many methods not limited to yeast display, structural biology techniques of cryogenic electron microscopy, nuclear magnetic resonance and X-ray crystallography, binding and neutralization assays, identification and investigations of escape mutants, the methods inform different aspects of antigen–antibody interactions and structural biology techniques can provide unique insights into quaternary epitopes. Secondly, current data show that, especially for B cell epitopes, sequence conservation of an epitope may not be sufficient to predict binding and/or neutralization, emphasizing a need for independent resolution of the underlying mechanisms of neutralization for different viruses [170]. For instance, E16 is a potent WNV antibody. Based on yeast display, critical residues and regions for antibody interaction were initially identified [172], the interactions were subsequently confirmed using structural studies [173]. However, substitution of a few residues within these regions renders E16 ineffective for USUV SAAR-1776 neutralization and shows an altered pattern for USUV CAR-1969 [75,174]. Polyclonal antibodies generated against WNV in humans have been reported to show narrow-specificity targeting regions in DI and DII rather than DIII, with the majority displaying broad cross-reactivity [168,170]; whether this is a feature of all members the JE serogroup including MVEV and USUV is unclear.

Flavivirus neutralization is affected by many factors, not all of which are well defined for a virus/strain. For instance, neutralization by mouse monoclonal antibodies (MAbs) against DENV4 was shown to be strain- and genotype-dependent; this neutralization varied at different temperatures (37 °C or 40 °C), whereas after incubation at 37 °C, cross-reactive antibodies against FL of DENV1–3 weakly neutralized multiple strains of DENV4 [127]. Known factors that affect binding and neutralization include accessibility of the epitope, affinity of the antibody, maturation and conformational state of the virus and the specific stoichiometry of the antibody to achieve neutralization [175,176]. Sub-neutralizing antibodies may lead to neutralization at higher concentrations [177]. The cryo-EM structure of WNV virus in complex with the therapeutic antibody E16 showed that the antibody targets sixteen residues on loops of DIII and forms a network of hydrogen bond interactions [173,178]; E16 was shown to neutralize by blocking acid-induced membrane fusion. In a separate study, two potent antibodies against JEV, 2F2 and 2H4, were also shown to bind quaternary epitopes; the binding interface spans three adjacent E monomers in the asymmetric subunit, with four such interfaces locking the E monomers of a raft [179]. On the E protein, the interfacing residues map to DIII and DI-DII hinge regions, which tend to differ between flaviviruses but are conserved among the genotypes of JEV. The two antibodies blocked receptor attachment and JEV entry as well as endosomal membrane fusion.

### 4.3. Flavivirus Cross-Reactivity

The impact of flavivirus cross-reactivity is pertinent for the development of therapeutics because of the presence of multiple flaviviruses circulating in any given region and hence the possibility of simultaneous or sequential (homotypic and especially secondary heterotypic) human infections. The changing geographic distribution of flavivirus strains/genotypes is in part due to changing global climate, as well as new introductions [180]. Complexity in the adaptive immune control of sequential, secondary homo- or heterotypic flavivirus infections results from: (1) Amino acid sequence conservation within the antigen affecting antigenic diversity. The highly cross-reactive FL of flavivirus E protein is also one of the most conserved regions across flaviviruses and mutations in this region result in lower cross-reactivity in in vitro studies [4,181,182,183]; (2) the original antigenic sin, which means that the secondary exposure to a variant, non-identical antigen is not recognized as such by the B cells (or the cytotoxic T lymphocytes) and the immune system relies on its ‘memory’ of the original, primary antigen to mount a response to the variant, which is inadequate and ineffective. This altered memory recall results in production of sub-neutralizing antibodies that fail to control the secondary infection or additionally leads to a worse clinical outcome [184]; (3) antibody-dependent enhancement (ADE), resulting from the original antigenic sin. When less effective, non-neutralizing antibodies are generated in response to an often heterotypic secondary flavivirus infection. These antibodies can be internalized and sequestered into monocytes, macrophages and mast cells containing fragment γ receptors (Fcγ) and complement receptors. This amplifies viral replication and leads to the worsening of disease. ADE is a hallmark of DENV and various flavivirus infections, including those involving vaccination-induced immunity in human infections and mouse models [185,186]. Antibody-facilitated infection enhancement via routes not involving cellular receptors has been shown in experiments and can also occur when antibody-bound infected cells are lysed by cytotoxic natural killer cells, referred to as antibody-dependent cellular cytotoxicity [185,187,188,189,190,191].

Cross-reactive immune responses to flavivirus infections are, therefore, a double-edged sword both for flavivirus diagnostics as well as therapeutics. While epitopes on non-structural proteins elicit adaptive immune responses, the neutralizing humoral response against the structural E protein constitutes a dominant avenue of protection against flavivirus infections; these antibodies are primarily directed toward epitopes localized on DIII and the lateral ridge and tend to be type-specific [169]. In vitro assays showed greater cross-reactivity and neutralizing titers when E protein was used as a marker rather than NS1, although titers of the neutralizing antibodies may not directly correlate with protection in natural infections [192]. In mouse experiments with WNV, DIII-epitope-generated antibodies were shown to comprise a fraction of the total initial antibody response and were overshadowed by cross-reactive, sub-neutralizing antibodies [193]. The latter can confer protection at higher concentrations or via the antibody effector functions involving complement fixation and antibody-mediated cytotoxicity, emphasizing the importance of B cell memory recall; therefore, lack of or partial neutralization in vivo may not be an ideal indicator of protection for outcomes for natural secondary infections [177,194,195]. Cross-reactive B cell epitopes are often characterized by distinct structural attributes such as cryptic presence on E and localization on the DII domain, near the FL, and these epitopes include distinct, highly conserved amino acid residues; peptide fragments in DII; and distinct as well as overlapping regions in the E protein, not limited to amino acid residues of the fusion loop [171,192,196,197]. Cross-reactive T cell epitopes associated with CD8 and CD4 T cells have been localized to the non-structural proteins as well as the E protein and induce either cross-protection or immunopathology in homotypic as well as heterotypic infections involving JE serocomplex viruses [4,198]. Because these epitopes elicit heterotypic cross-protection, there’s a need to define the molecular and immunological determinants of cross-reactive T cell-based immunity in animal models that can be used to inform the development of a pan-flavivirus or pan-JE-serocomplex-virus vaccine [199,200,201].

Pre-existing immunity to a flavivirus can result from asymptomatic viral exposure, natural viral infection or immunization. Four licensed vaccines for human use exist for JEV infection (Table 3) and vaccines for human use are unlikely to be developed for other JE serocomplex viruses that cause infrequent outbreaks. These licensed vaccines and those under development were utilized to explore cross-reactive immune responses in animal experiments. JEV vaccination using JE-ADVAX or a DNA vaccine derived from the expression of prM/E proteins induced protection against a lethal JEV challenge in animal experiments, resulting primarily from humoral and cellular immunity while the CD8 T cell immunity was dispensable for survival [161,202,203]. Similar experiments in mice using recombinant vaccinia virus that carried the genes for the E and NS1 proteins of MVEV showed complete protection with a subsequent challenge with MVEV by eliciting generation of neutralizing antibodies, rather than a CD8+ immune response when E protein was used; passive transfer of MVEV-infected human sera also conferred protection with a subsequent MVEV challenge [204]. Cross-protection due to neutralizing antibodies resulting from CD4 T cell expansion following infection after JEV immunization, but not with YF immunization, has also been reported against ZIKV and DENV [205,206,207]. Li et al. reported cross-protection against all four serotypes of DENV following JEV immunization, using more than one JEV vaccine [208]. Chimeric vaccines using components of the JEV live vaccine strain SA-14-14-2 have been reported to protect against YF and TBEV infections, with dual protection against YFV and JEV in case of the former [209]. Determinants of cross-protection via CD4 T cell immunity map to two helices in the capsid protein and regions of E [210,211]. Koblischke et al. [210] reported peptide regions that form the immune-dominant WNV E epitopes, and one such region (E149) is unique and absent in DENV and ZIKV and lies on a structurally divergent region in WNV. Multiple research findings from mouse experiments and human studies revealed the essential role of cellular immunity and neutralizing antibodies in cross-protection between primary JEV or WNV exposure and secondary DENV and ZIKV infections [212]. The sequence of infection is known to influence disease outcomes; additionally, immune-dominant mechanisms of protection for each dyad of viral infections can be distinct and essential to elucidate if these outcomes are to inform therapeutics. For instance, in the sequential JEV-ZIKV infection, unlike the JEV-JEV infection, JEV vaccination-induced CD8 T cell immunity was found to be essential for conferring cross-protection in mouse models compared to passive transfer of serum [213,214]. Alternatively, cross-protective outcomes in JEV-DENV1 infection in mouse models required the cooperative effects of humoral and T cell responses, whereas in human infections, secondary DENV following anti-JEV immunity resulted in manifestation of increased viral symptoms [206,212,215].

Multiple studies explored the outcomes of sequential homotypic infections with JE serocomplex viruses (Table 3 and Table 4). Vaccination of different mouse models with JEV (JE-ADVAX, live-chimeric JEV vaccine) or sera from adult mice that were infected sub-lethally with JEV revealed the generation of cross-protective humoral and cellular immunity and the protection of homotypic secondary MVEV and WNV infections [195,216,219,221,222]. In some studies, protection was observed in the absence of detectable neutralizing antibodies and dispensable CD8 T cell immunity, emphasizing the role of memory B cells in conferring long-term protection against the secondary MVEV or WNV infections virus [195]. Animal studies in macaques revealed that, while immunization with JEV vaccine completely protected against a secondary WNV infection, immunizing with the latter protected partially against disease severity [217]. These studies emphasize the potential of using JEV vaccines against a range of JE serocomplex viruses. In the human population in Europe, USUV antibodies have been found in patients with severe WNV neuro-invasive disease [235]. Blazquez et al. [229] reported differential susceptibilities of adult and suckling mice to USUV infection, neutralization of secondary USUV following WNV infection and protection against neuro-invasive WNV disease in mice pre-infected with USUV. WNV recombinant subviral particles were able to induce cross-reactive humoral response in USUV, albeit at low levels, and a USUV-based recombinant DNA vaccine could also elicit neutralizing antibodies in adult mice deficient in interferon alpha/beta receptor [227,233].

Understanding the factors that drive disease enhancement in sequential JE serocomplex viruses is essential to design therapeutic interventions for multiple viruses and because human outbreaks of some JE serocomplex viruses are too infrequent to support development of virus-specific vaccines. Experimental studies [220] caution the possibility of ADE when the effect of sub-optimal concentrations of neutralizing antibodies against JEV were used to explore the effect on a secondary infection with MVEV. Similarly, immunization with MVEV, using killed virus or vector-delivered structural-protein-based subunit vaccine, followed by challenge with JEV in BALB/c mice showed enhanced disease and protection, respectively [186,220]. A low dose of inactivated JEV vaccine or sera from mice that were sub-lethally infected with MVEV caused ADE when subsequently challenged with MVE. However, in a different study, utilizing inactivated JEV vaccine in animals elicited partial protection against WNV infection, emphasizing the importance of consideration of dosage of the priming virus [216,221]. Sub-lethal immunization with KUNV instead of WNV and passive transfer of sera of mice infected with MVEV resulted in enhanced secondary MVEV disease [216]. However, disease enhancement in the human population in response to a JE vaccine eliciting humoral response with or without augmenting T cell immunity is seemingly unlikely [194,220,243] and the robust immune responses generated to cross-protect JE viruses suggest a strong consideration for harnessing cross-protective immunity for vaccine development. 

## 5. Summary

The end goal of flavivirus research centers on developing vaccines and effective therapeutics. With the climate crisis and changing distribution of vectors and epidemiology of pathogens, this need is urgent. To this end, convergence of research on drivers of epidemiological changes, flavivirus structural biology, studies in animal models and natural exposure in humans, and immunology can advance our understanding. Connecting the dots between immunological correlates of cross-protection with molecular mechanisms and interactions is challenging, and is limited, in part, by the resolution and availability of structures of complexes of virus and host components. Recent high-resolution structures of JE serocomplex flaviviruses, advances in methods in cryo-electron microscopy and deep learning approaches may enable greater understanding to design effective strategies in the near future. 

## Figures and Tables

**Table 1:** Listed are some vectors and animal hosts of the four viruses from Japanese encephalitis serocomplex and the clinical symptoms in human infections.

Virus	Mosquito Vectors	Natural Reservoir/Amplifying Hosts	Animal Hosts/Animals Infected	Human Clinical Symptoms	References
JEV	*Culex* spp. *including* *Cx. tritaeniorhynchus* *Cx. vishnui* *Cx. gelidus* *Cx. annulirostris* *Cx. annulus* *Cx. fuscocephala* *Cx. sitiens* *Cx. quinquefasciatus* *Cx. bitaeniorhynchus; Aedes, Anopheles, Mansonia and Armigeres* spp. *including * *Aedes albopictus*	Ardeidae family such as egrets, herons; ducklings, chicken, American crow, house finch, house sparrow, ducklings	Feral and domestic pigs, horses, boars, racoons, dogs, bats, cattle, goats	Febrile illness without any other clinical manifestation; acute encephalitis including headache, vomiting, seizures; flaccid paralysis, facial paralysis, hepatomegaly, splenomegaly, Thrombocytopenia, Guillain Barré syndrome, neurological sequelae	[8,9]
MVEV	*Cx. annulirostris,* *Cx. australicus* *Aedes normanensis* *Ochlerotatus tremulus*	Ciconiiformes such as herons, egrets	Kangaroos, rabbits, mice, dogs, pigs	Febrile illness, encephalitis, neurological sequelae, flaccid paralysis	[10]
WNV	*Culex spp. including* *Cx. tarsalis* *Cx. quinquefasciatus* *Cx. stigmatosoma* *Cx. thriambus* *Cx. pipiens* *Cx. nigripalpus*	Corvidae family such as American Crows and blue jays, common grackles, house sparrows, American robins, house finches	Rabbits, lemurs, hamsters, squirrels, chipmunks	Fever, myalgia, encephalitis, meningo-encephalitis, meningitis, flaccid paralysis	[11,12]
USUV	*Culex* spp. *Including* *Cx. pipiens* *Cx. neavei* *Cx. modestus* *Cx. perfuscus* *Cx. quinquefasciatus * *Aedes, Anopheles Mansonia and Ochlerotatus* spp. *Aedes albopictus* *Aedes japonicus*	Passeriformes (such as Eurasian blackbirds, house sparrows and Magpies), Strigiformes (such as Great grey owl), Coraciiformes	Horses, bats, dogs, cattle, wild boar, deer, rodents, shrew	Fever, skin rash, meningo-encephalitis, facial paralysis Asymptomatic infections/presence in donated blood from healthy adults	[13,14]

**Table 2:** Listed are the lineages of JEV, MVEV, WNV and USUV with a few representative strains, their geographical distribution and disease incidences.

Virus Lineage	Alternative Name/Included Strains	Accession Numbers	Geographical Distribution	Disease Prevalence	References
**JEV**					
Genotype I	KV1899	AY316157	Asia including Korea, China, Japan, Cambodia, Vietnam, Thailand	Epidemics in temperate regions, predominant in humans	[8,9,29]
	Ishikawa	AB051292			
	HEN0701	FJ495189			
Genotype II	FU	AF217620	Asia, Australia, Korea	Endemic disease in tropical regions	[8,9,29]
Genotype III	p3 SA14-14-2 Vellore P20778	U47032 AF315119 AF080251	Asia including India, China, Japan, Korea (Temperate zones)	Epidemics in temperate regions, endemic activity, predominant in humans	[8,9,29]
Genotype IV	JKT6468	AY184212	Indonesia and Australia	Endemic disease in tropical regions	[8,9,29]
Genotype V	Muar strain XZ0934	HM596272 JF915894	Asia including Malaysia, China and Korea	Re-emerged strain, Human encephalitis	[8,9,29,30]
**MVEV**					
Genotype 1	MVE-1-51 K49077 08-154300	AF161266 EF015056 JN119766	Australia	Epidemics, sporadic cases	[31]
Genotype 2	OR156 K6454	EF015074 EF015070	Australia	Human encephalitis	[32]
Genotype 3	NG156	EF015076	Papua New Guinea	Human encephalitis	[10,33,34]
Genotype 4	MK6684	EF015075	Papua New Guinea	-	[31]
**WNV**					
Lineage I	HNY1999 VLG4 LEIV-Vlg99-27889 Eg101 Kunjin MRM61C	AF202541 AF317203 AY277252 AF260968 D00246	Europe, Russia, North Africa, Israel, United States, Middle-east, Australia and India	Outbreaks in humans	[12,35,36,37]
Lineage II	Sarafend Ug37	AY688948 NC_001563	Sub-saharan Africa including South Africa, Madagascar; Europe including Greece, Russia	Zoonotic outbreaks in South Africa, avian and human outbreaks in Europe	[12,35,37,38]
Lineage III	Rabensburg virus (RABV/97-103)	AY765264	Europe	-	[35,36,39]
Lineage IV	LEIV-Krnd88-190	AY277251	Russia	-	[36]
Lineage V	804994 G16146	DQ256376 GQ851605	India	Outbreaks in humans	[12]
Lineage VI	HU2925/06	GU047875	Spain	-	[40]
Lineage VII	Koutango virus ArD96655	KY703855	Africa, Malaysia, Senegal	Sporadic outbreaks in Africa	[37]
Lineage VIII	ArD94343	KY703856	Senegal	Sporadic outbreaks in Africa	[37]
Lineage XI	WNV-Uu-LN-AT-2013	KJ831223	Austria	-	[37,40]
**USUV**					
Africa 1	Central African Republic 1969 (CAR 1969)	KC754958	Africa	-	[13,41,42]
Africa 2	South Africa1959 (SAAR 1776) Spain 2006	AY453412 KF573410	Africa Europe	-	[13,41,42]
Africa 3	Central African Republic (CAR 1981) Senegal 2007 Netherlands 2016	KC754955 KC754957 KY128482	Africa Europe	Human illness	[41,43]
Europe 1	Austria 2001 (Vienna 2001) Hungary 2005	AY453411 EF206350	Europe	Human meningo-encephalitis	[13]
Europe 2	Italy 2009 Austria 2016 Hungary 2016	HM569263 MF063042 MF063043	Europe	Human meningo-encephalitis, meningitis, asymptomatic blood donors	[42,44,45]
Europe 3	Germany 2011 Belgium 2016 France 2016	KJ438769 KX977447 KY128481	Europe	Asymptomatic blood donors	[13,42]
Europe 4	Italy 2009 Italy 2010	HM138711 JF834562	Europe	Asymptomatic blood donors	[13,42]
Europe 5	Germany 2016 France 2016	KY113091 LT854220	Europe	Atypical human illness	[13,14]

**Table 3:** Cross-reactivity among the four JE serocomplex viruses, JEV, MVEV, WNV and USUV, and the outcomes for disease are depicted. Viruses listed across represent preexisting immunity, primary challenge or vaccination and viruses down the column represent secondary infection. Flaviviruses circulating in distinct areas with no currently reported co-existence are denoted by ‘Distinct geography’.

Virus (Infection or Vaccination)	Outcome	JEV	MVEV	WNV	USUV
JEV	Protection Pathology	[203] [195] -	[216] [186] [217] [218] [186]	[203] [217] [219] [220]	Distinct geography
MVEV	Protection Pathology	[221] [217] [195] [186]	[216] [204] -	TBD/Unknown TBD/Unknown	Distinct geography
WNV	Protection Pathology/no protection	[222] [223] [195] [224] [217] [219] [225] [226]	TBD/Unknown -	[227] [228] -	[229] [230] [231] -
USUV	Protection Pathology	Distinct geography TBD/Unknown	Distinct geography TBD/Unknown	[227] [232] TBD/Unknown	[233] [234] -

**Table 4:** Vaccines for JE serocomplex viruses that are licensed for use in humans.

Virus	Strain	Vaccine	Platform	References
JEV	Genotype III	JE-VAX	Inactivated, derived from mouse brain	[236,237,238]
JEV	Genotype III	SA-14-14-2	Live attenuated strain SA-14-14-2, derived from cell culture	[239]
JEV	Genotype III	IXIARO, JEBIK V	Inactivated, derived from cell culture	[240,241,242]
JEV	Genotype III	IMOJEV	Attenuated, chimeric, derived from cell culture	[241]
